# Cephalopod body size and macroecology through deep time

**DOI:** 10.1038/s41598-025-13940-1

**Published:** 2025-08-21

**Authors:** Christian Klug, Dirk Fuchs, Alexander Pohle, Dieter Korn, Kenneth De Baets, René Hoffmann, David Ware, Peter D. Ward

**Affiliations:** 1 Paläontologisches Institut und Museum, Karl-Schmid-Strasse 4, 8006 Zürich, Switzerland; 2 Bayerische Staatssammlung Für Paläontologie Und Geologie, Richard‑Wagner‑Straße 10, 80333 Munich, Germany; 3 Institut für Geowissenschaften, Ruhr-Universität Bochum, 44801 Bochum, Germany; 4 Museum für Naturkunde, Leibniz Institute for Research on Evolution and Biodiversity, Invalidenstraße 43, 10115 Berlin, Germany; 5 Institute of Evolutionary Biology, Faculty of Biology, University of Warsaw, ul. Zwirki i Wigury 101, 02-089 Warsaw, Poland; 6 Department of Biology, University of Washington, Seattle, WA 98995 USA

**Keywords:** Ammonoida, Neocoleoidea, Nautilida, Phanerozoic, Gigantism, Macroecology, Palaeoecology, Palaeontology

## Abstract

As actively swimming predators, cephalopods have played a key role regulating and engineering marine ecosystems for more than 500 million years and continue to do so. For the first time, we portray fluctuations of cephalopod body size including species from the Cambrian to today. For comparability, we determined the maximum body volumes of each species using various proxies, because classical measures like mantle length cannot be applied homologously to all groups. We separately examined Cephalopoda with orthoconic conchs (without ammonoids and coleoids), Nautilida, Ammonoida (without heteromorphs), and Neocoleoida (squids, octopuses and their ancestors). The long-term trajectories of these groups differ in their overall trends. Each of these groups reacted in other ways to the mass extinctions. All groups except the nautilids evolved species exceeding one meter in size, which belong to the marine megafauna. Nautilids and orthocones share a threshold volume of about 100 litres, while in ammonoids and neocoleoids, the limit lies closer to 500 litres.

**Supplementary Information:**

The online version contains supplementary material available at 10.1038/s41598-025-13940-1.

Throughout their half-billion-years of evolutionary history, cephalopods have been and continue to be vital components of global marine food webs^1^. Their respective ecological position ranges from microphagous predators^2–6^, through mesopredators^7–9^ to higher trophic positions or possibly apex predators in marine food webs^10–15^. Their locomotory abilities rely on both their body size and their anatomy; a nektic habit depends on the Reynold’s number exceeding 100, which corresponds to a body length of over 10 cm^15^, but there are exceptions such as some fish as small as 15 mm^15^, which can still be considered nektic (e.g., actively swim against currents). In turn, huge orthocones such as the Ordovician endoceratids^5,16^ were possibly not as actively swimming as their huge body size suggests^17,18^, while most post-Palaeozoic neocoleoids (all endocochleates starting from the donovaniconids via belemnites to the decabrachians and octobrachians) were likely good swimmers^19,20^. Among ammonoids, swimming abilities varied and depended not only on body size, but also on conch geometry^20–24^.

Body size matters for cephalopods as it does for all other organism groups. In metazoans, it often increases through the evolution of a clade^25,26^. Body size correlates with metabolism (Kleiber’s Law^27^) and accordingly with population size as well as the position in food webs^28,29^. In marine environments, this relationship is sometimes less distinctly developed, for example among filter feeders^30–32^. The largest cephalopods that exceed a length or diameter of one meter have been considered marine megafauna by Pimiento et al.^26^.

Fluctuations of cephalopod sizes through deep time have rather rarely been studied^33^, although it was discussed in the context of Cope’s rule among ammonoids^18,34,35^ or polar gigantism^17,36^. Stevens^35^ provided one of the few surveys of ammonoid size evolution through the Mesozoic. His research interest was likely triggered by the discovery of a giant lytoceratid in New Zealand^37^. But even this huge Late Jurassic ammonite with a conch diameter of 1.5 m does not reach the long-standing world-record of *Parapuzosia seppenradensis* with its preserved diameter of 1.74 m (Fig. 1) and a reconstructed diameter of 2.05 m^38,39^. Unverified reports suggest that the northern outcrops of Campanian strata in Mexico bear even larger individuals of the same genus, but proof thereof is still lacking^40^.

The largest cephalopod fossils currently known are Ordovician endoceratids. Although fragmentary, the 3 m long conch part in the collection of the Museum of Comparative Zoology at Harvard University figured by Teichert & Kummel^41^ is currently the largest cephalopod fossil we are aware of. In the same article, they report a 10 m long specimen, but this was neither rescued nor photographed. Another specimen of similar size as the specimen in Harvard is on display at the University of Michigan (Fig. 1). We reconstructed the conch size of the Museum of Comparative Zoology at Harvard University specimen, which was extrapolated to a length of 5.73 m^17^. Note that this reconstructed length assumes a body chamber length of 30% of the total length. The proportion of the body chamber in endoceratids is poorly constrained, but from the few known specimens with complete body chambers, it appears to have been comparatively short^43^, i.e. perhaps less than 30%. A report of a 10 m long specimen^42^ is commonly cited as the known length attained in the genus “*Cameroceras*” (the taxonomic status of this genus needs clarification) in popular media. However, there exists neither physical evidence nor a photograph of this specimen^42^. As this size is more than triple the length of the longest confirmed specimens, this report remains doubtful. We stress that the generally gigantic size of endoceratoids is a misconception – the vast majority is considerably smaller^42,43^. Other orthocones, usually assigned to the actinocerids^18,44^, have reconstructed conch lengths of 2.6 m (Emsian *Deiroceras*^17,18,44^ and 2.8 m (Carboniferous *Rayonnoceras*^45^. Their body volumes, particularly of the soft parts, were small compared to, e.g., those of the largest ammonoids or neocoleoids.

From a modern-day point of view, giant squids come to mind when considering cephalopod size. These impressive animals have triggered human imagination for centuries, especially because the largest representatives are quite elusive due to their deep marine habitat^46^. It was not until twenty years ago that living individuals have been filmed in the wild^47^. While the heaviest squid ever recorded is *Mesonychoteuthis hamiltoni* with 490 kg^48^, which is also the heaviest known invertebrate^49^, the giant squid *Architeuthis dux* is the longest. In social media, bold speculations about its maximum size range up to 20 m, which roots probably in the fact that the long tentacles are flexible and when pulled, they can reach such lengths; the verified and accurately measured maximum length of the giant squid including tentacles is 12 m^49,50^. In contrast to the decabrachians (ten-armed neocoleoids), the largest octobrachians (eight-armed neocoleoids) are much smaller with *Enteroctopus dofleini* with a recorded arm span of 6 m and a maximum body mass of 50 kg^51^, while the seven-arm octopus *Haliphron atlanticus* may attain a slightly bigger body mass of up to 75 kg^52^ although it has much shorter arms. Such data already demonstrate how uncertain body mass and size estimates can be, even when recent species are considered^33^.

In fossil taxa, estimating the maximum size depends on the group and the completeness of the fossils (e.g., deformation or lack of soft tissues). While in some groups body length corresponds to skeleton length, these values may differ significantly in invertebrates including cephalopods. We herein focus on the skeleton length and volume as it is most consistently known over time and most comparable to size metrics used for modern cephalopods. If one adds the wishful thinking of having discovered the largest specimen or species of a group, the great biases in size estimates become evident. Recently, these issues were summarized for some iconic gigantic fossil species including some cephalopods^53^. An additional issue is comparability. Taking ectocochleate cephalopods (with external conch) into account, forms with straight conical conchs (orthocones) attained impressive lengths but probably had lower volumes and body mass than, e.g., some ammonoids with coiled conchs.

**Fig. 1:**
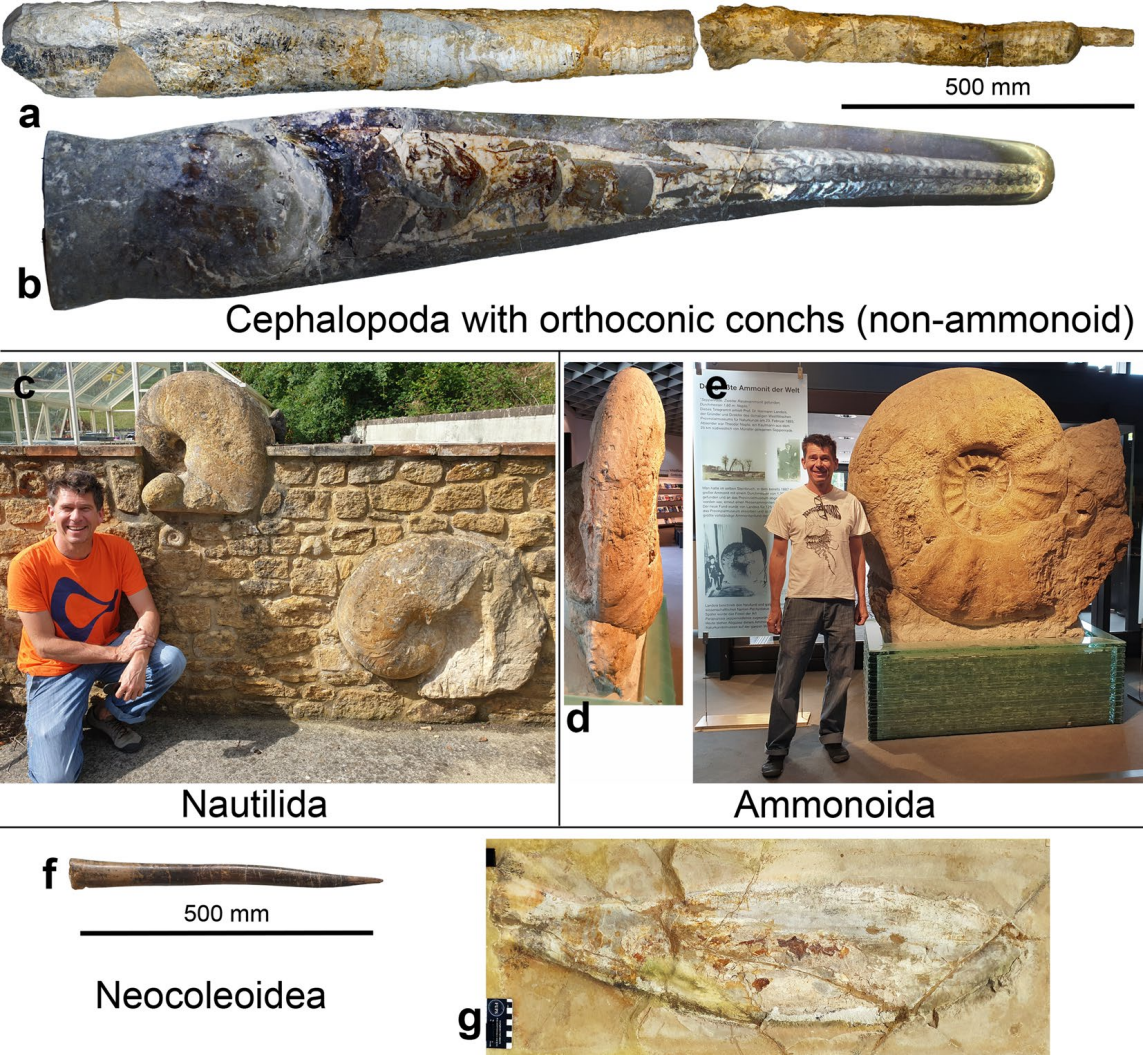
Some of the largest known cephalopods as examples for the four studied groups. **a**, *Endoceras giganteum*, UMMNH 2019.0385, Platteville, Illinois (display in the University of Michigan Museum of Natural History). **b**, *Deiroceras hollardi*, PIMUZ 31922, Early Devonian, Jebel Mdouar, Morocco (display in the Museum of Natural History, University of Zurich). **c**, *Cenoceras rumelangense*, Bajocian, Dorset, UK (W. Grulke collection). **d**, ventral and **e**, lateral view of *Parapuzosia seppenradensis, *Campanian, Seppenrade, Germany, displayed at the LWL museum in Münster. f, *Megateuthis elliptica*, SMNS 60752, Bajocian, Bopfingen-Oberdorf; Germany (display at the Staatliches Museum für Naturkunde, Stuttgart). **g**, *Leptotheutis gigas*, Tithonian, Solnhofen (display in the Schaulager Ruhrmuseum, Essen). All photos except a belong to CK. Photo in a by courtesy of J. Bauer (University of Michigan Museum of Natural History).

Here, we provide the first attempt to portray the body size evolution of four main groups of cephalopods through deep time from their late Cambrian origin to today. We analyse each of the groups separately using literature data and specimens we examined ourselves in various collections. For comparability, we use both lengths/ diameters and body volumes (the entire animal including conch and arms), the latter being better comparable between different cephalopod bauplans. Our research questions are: (i) Is maximum body size evolving randomly, is it statistically constant through time in each group and in the whole group or, if not, how does it change? (ii) How did the mass extinctions affect body size? (iii) Are there other macroecological processes that influence body size?

## Results

### Main trends

**Fig. 2:**
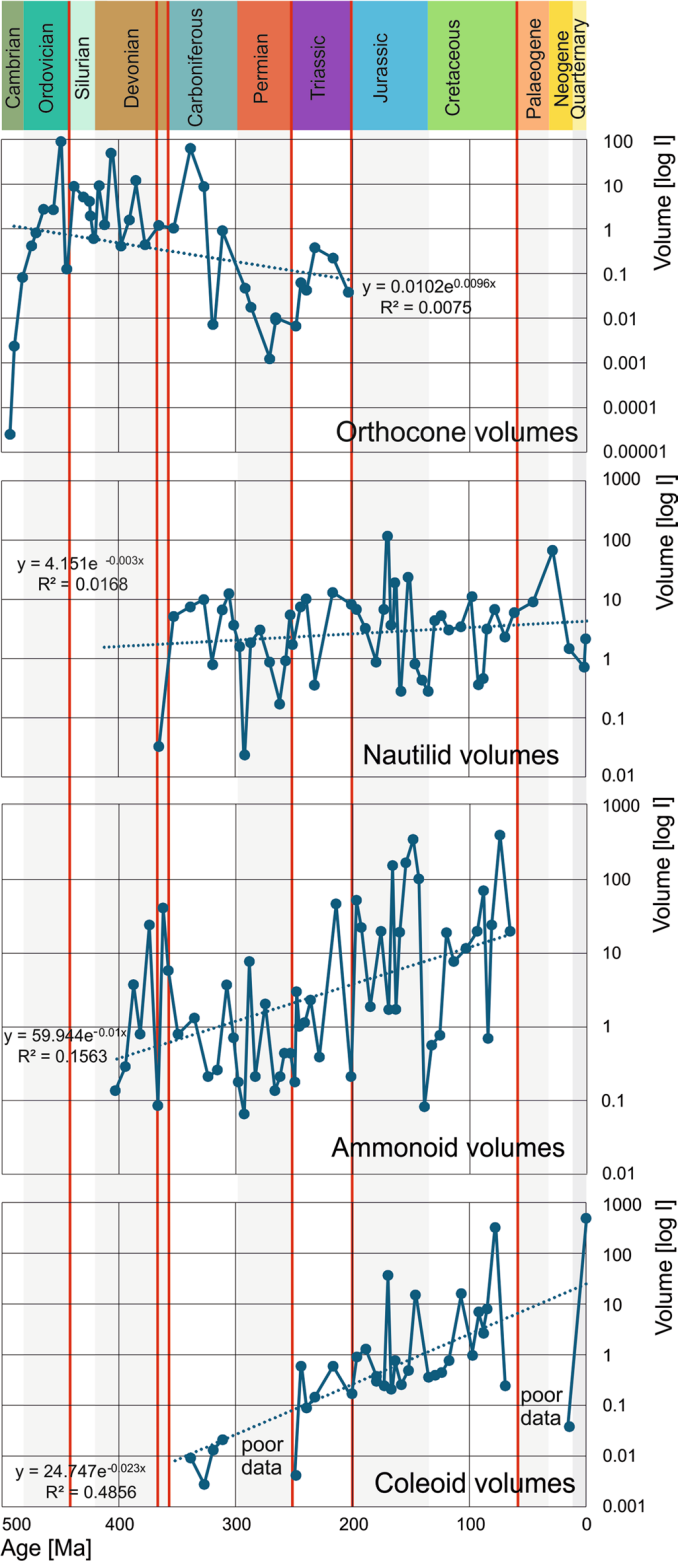
Volumes of the largest representatives per stage of the orthocones, nautilids, ammonoids and neocoleoids. The trendlines are the best fitting exponential trendlines, volumes are given in log litres. Red lines represent mass extinctions.

Following an initial size and volume increase by several orders of magnitude, most of the largest representatives of the four groups (orthocones, nautilids, ammonoids, neocoleoids) share a maximum total conch volume between four and ten litres (Fig. 2). In the orthocones, the steep initial size and volume increase is followed by a plateau in the Ordovician until the Viséan and followed by a subsequent decline during the Permian and a small peak in the Triassic before their demise. The low values in the Permian might root in poorer sampling.

**Fig. 3:**
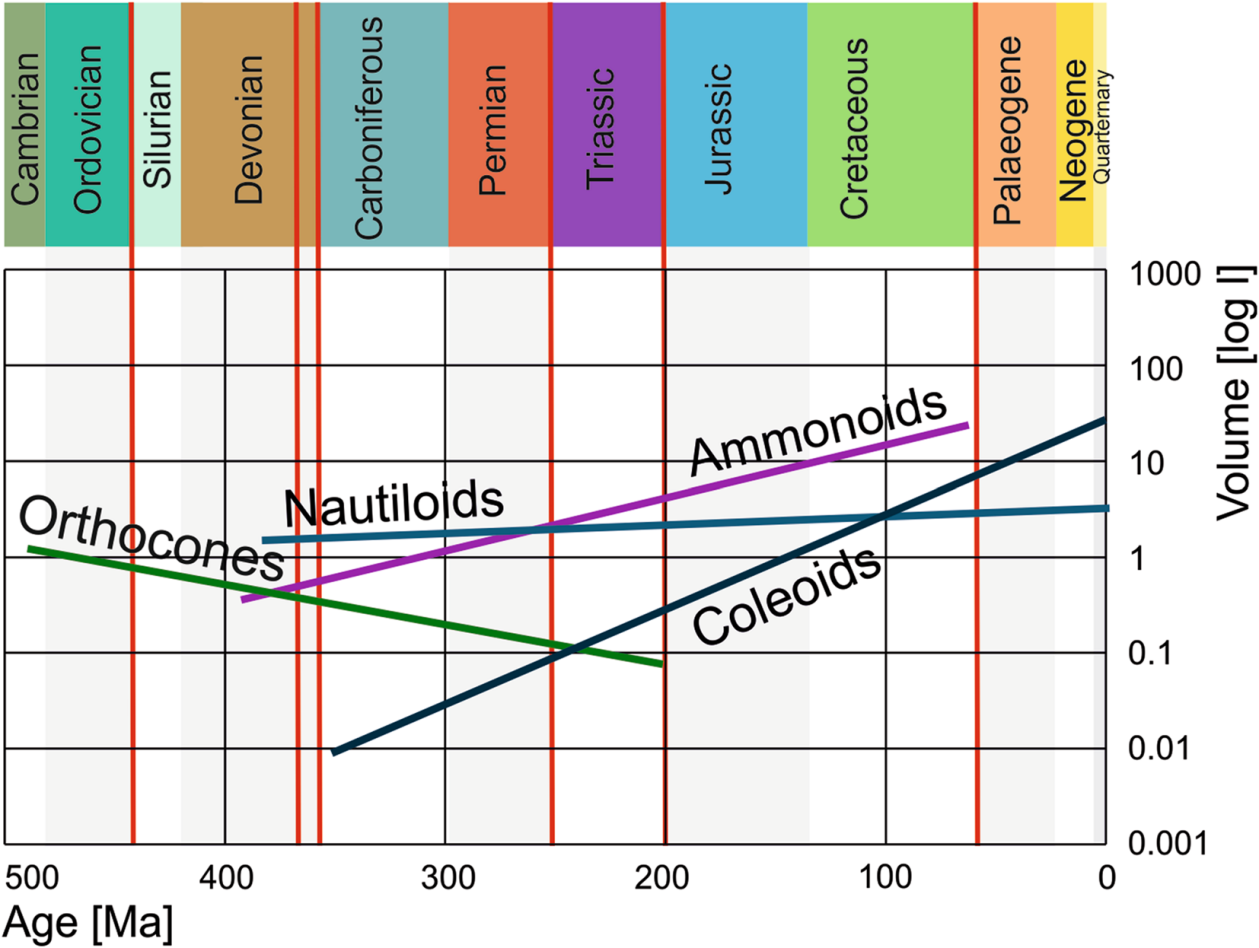
Exponential trendlines of the volumes of the four examined cephalopod groups through the Phanerozoic. Horizontal bars display the duration of the four groups. Red lines represent mass extinctions.

Nautilids display the most consistent maximum conch sizes and volumes across the Phanerozoic, showing only a minimal overall increase over time since their appearance in the Devonian (Fig. 3). The size and volume fluctuations appear to be lower than in the other groups. Maintaining similar body sizes may be related to low turnover rates and conservative morphology over long time intervals. Perhaps, their low metabolism^54,55^ depends on a maximum body size that can be maintained with the feeding strategy as suggested by Kleiber’s law^27^.

**Fig. 4:**
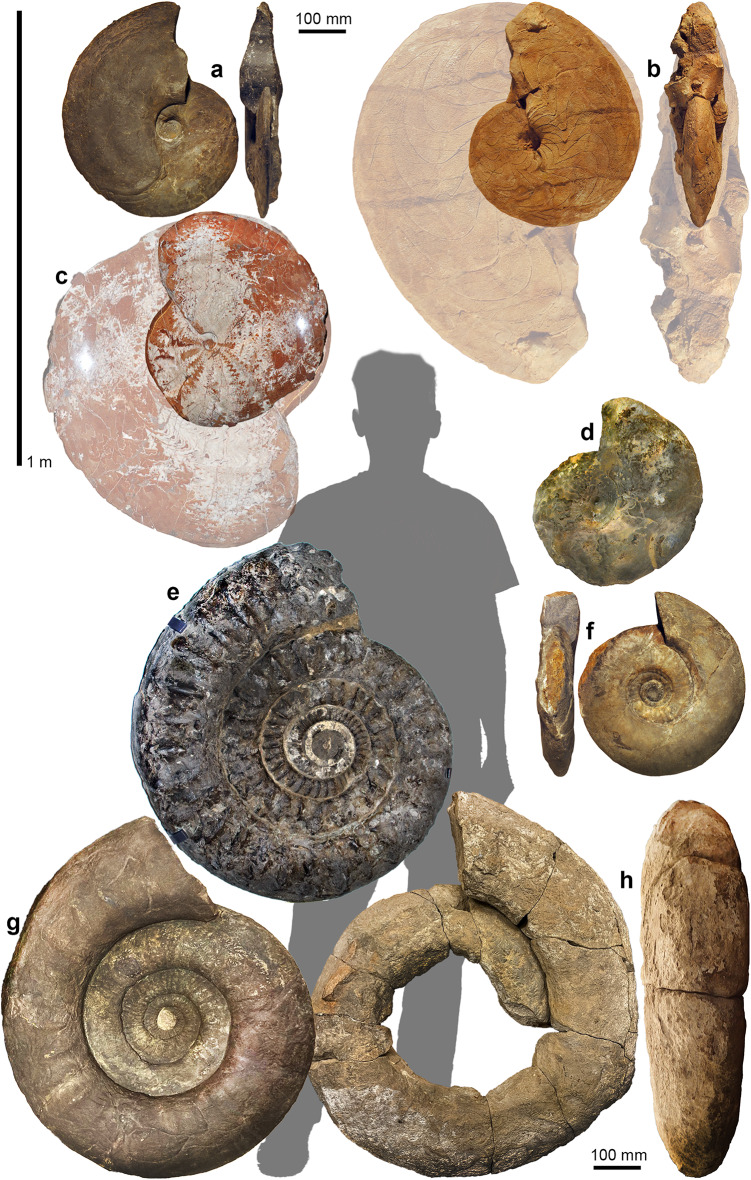
Some examples of ammonoids that are among the largest of their times (silhouette of a 1.8 m tall person). **a**, *Agoniatites expansus*, PIMUZ 41294, late Eifelian, Aferdou El Mrakib, Morocco. **b**, *Manticoceras* sp., PIMUZ 41298, late Frasnian, Tafraoute Sidi Ali, Morocco. c, *Pinacoceras metternichi*, Norian, Feuerkogel, Austria (Naturhistorisches Museum, Vienna). **d**, *Discoceratites semipartitus*, MHI 1246/5, Ladinian, Dettelbach, Germany (Muschelkalkmuseum Hagdorn, Ingelfingen). **e**, *Arietites* sp., Sinemurian, Frick AG, Switzerland (Naturhistorisches Museum Basel). **f**, *Procerites quercinus*, PIMUZ 51303, Bajocian, Holderbank AG, Switzerland. g, *Binatisphinctes* sp., Be 39719, Callovian, Herznach AG, Switzerland (Naturhistorisches Museum Bern). **h**, Reineckeidae, MGL.101606, Callovian, Baulmes VD, Switzerland (Muséum cantonal des sciences naturelles, Lausanne).

Ammonoids are the best documented among the four groups, likely because of their wide use as index fossils and their high abundance in pelagic sediments (Fig. 4). The steep size and volume increase in the Devonian^56,57^ corresponds to the simultaneous morphospace expansion^58–60^. This was interpreted as a Red Queen effect^57^ during the evolution of jawed fish-predators and their ammonoid prey. After the Devonian, there was an extended phase where no exceptionally large ammonoids exceeding 0.6 m conch diameter are known (Carboniferous to Middle Triassic). Only in the Late Triassic, a first comparatively large ammonoid (*Pinacoceras*; Fig. 4c) evolved that fulfils the megafauna criterion^26^ and during the Jurassic and Cretaceous, different clades brought forth species with conch diameters of one meter or more^35^. Importantly, the largest ammonoid currently known is *Parapuzosia seppenradensis*, described 130 years ago^38^. This specimen is on display in the LWL museum in Münster (Fig. 1d). It misses part of the body chamber and measures 1.74 m in diameter and 2.05 m when reconstructed^39,40^. After the Campanian occurrences of the giant *Parapuzosia*, there were still large species of close to one meter in conch diameter shortly before the demise of the ammonoid clade^61^.

While the Permian and Triassic neocoleoids are in a similar volume range as orthocones, their body sizes and volumes rose quickly after the Triassic, a trend that continued until today. Overall, the trendline of the coleoid volumes is the steepest, followed by ammonoids (Fig. 3). Although the neocoleoids are likely monophyletic^62,63^, their morphology varies significantly, making it a heterogeneous group. Even when taking the biases in volume determination into account, there is a long-term increase in volume at least from the Triassic until today.

### What are the largest representatives of each group?

For the orthocones, it was the endoceratids that reached a conch length of at least close to five meters^17,41^. The largest representatives reached volumes of 100 L and accordingly a body mass of about 100 kg or more. It is noteworthy that large individuals can be found abundantly in different regions as far apart as the Baltic Sea and the New York region. This suggests that they accumulated an important biomass and thus played a key role in Ordovician oceans^5^.

Nautilids did not reach the megafauna threshold value at a diameter of up to 0.7 m. Remarkably, this corresponds to a similar volume of an endoceratid of 5 m conch length. Nautilids possibly did not have, because of lower metabolic rates, to exceed a conch diameter above 70 cm (cf. Kleiber’s law^27^.

Ammonoids attained conch diameters of about two meters or more^37,39^. The largest ammonoids might have reached volumes of about 400 L, which corresponds to a body mass of about slightly over 400 kg. This estimate is quite close to the heaviest modern squids such as the colossal squid (Table 1).

**Table 1:** Incomplete list of cephalopod species of megafauna size, i.e. With body size exceeding one meter^26^. Note that there are no nautilid species exceeding a conch diameter of 800 mm. Dimensions are conch lengths in orthocones, conch diameters in ammonoids, and mantle lengths in neocoleoids.

Group	Age	Species	Dimensions mm	Volume l
Orthocones	Ordovician	*Proterovaginoceras incognitum*	1000	0.8
Orthocones	Ordovician	*Ormoceras giganteum MB.C.11,940*	1710	2.72
Orthocones	Ordovician	*Ormoceras TUG 1308-1*	1720	2.66
Orthocones	Ordovician	*Endoceras giganteum*	4730	89.04
Orthocones	Ordovician	*Lambeoceras lambei*	1405	
Orthocones	Silurian	*Geisonoceras crebristriatum*	1911.41	8.9
Orthocones	Silurian	*Orthoceras gregarium*	1390	5.13
Orthocones	Silurian	*Temperoceras aequinudum*	1333	9.21
Orthocones	Devonian	*Deiroceras hollardi*	2001.83	48.91
Orthocones	Devonian	*Zeravshanoceras priscum*	1299.3	1.57
Orthocones	Devonian	*Basiloceras goliath*	1200	12
Orthocones	Devonian	*Plagiostomoceras* sp.	1100	0.029
Orthocones	Carboniferous	*Rayonnoceras solidiforme*	2800	62.51
Orthocones	Carboniferous	*Actinoceras vaughanianum*	1197.56	8.71
Ammonoids	Devonian	*Costaclymenia* sp.	1000	138.39
Ammonoids	Triassic	*Pinacoceras metternichi*	1040	154.21
Ammonoids	Jurassic	*Lytoceras*	1500	421.34
Ammonoids	Jurassic	*Lobolytoceras*	1540	452.92
Ammonoids	Jurassic	*Lytoceras taharoaense*	1500	421.34
Ammonoids	Jurassic	*Lytoceras*	1925	835.87
Ammonoids	Jurassic	*Corbinites occidentalis*	1320	296.61
Ammonoids	Cretaceous	*Emericiceras thiollierei*	?1000	
Ammonoids	Cretaceous	*Parapuzosia seppenradensis*	2050	934.75
Ammonoids	Cretaceous	*Diplomoceras maximum*	1025	148.09
Ammonoids	Cretaceous	*Baculites grandis*	?1500	?10
Neocoleoids	Jurassic	*Megateuthis elliptica*	1360	31.7
Neocoleoids	Jurassic	*Megateuthis suevica*	1000	36.61
Neocoleoids	Jurassic	*Leptoteuthis gigas*	1300	28.79
Neocoleoids	Cretaceous	*Eromangateuthis soniae*	1200	24.54
Neocoleoids	Cretaceous	*Enchoteuthis melanae*	2000	88.25
Neocoleoids	Recent	*Mesonychoteuthis hamiltoni*	3000	495
Neocoleoids	Recent	*Onykia robusta*	2000	41.73
Neocoleoids	Recent	*Architeuthis dux*	2690	275

As mentioned above, the body volume of neocoleoids shows the strongest increase and thus has the steepest trendline from their origin until today. The largest fossil coleoid *Enchoteuthis melanae* is from the Campanian like the largest known ammonoid. Its mantle length was given as two meters^11,13^. This corresponds to an estimated body volume of about 320 L and a similar amount in kilograms of body mass. Consequently, the Cretaceous neocoleoids had already reached a comparable size, volume, and mass range as their modern relatives.

Marine megafauna was defined by Pimiento et al.^26^ as species exceeding an adult body size of one meter. This criterion is fulfilled only by a few species in three of the four groups (Table 1). Only nautilids never reached such a conch diameter. In orthocones, representatives of the Ordovician endoceratids as well as Devonian and Carboniferous actinocerids fall in the megafauna size range. The first ammonoid reaching this size might have been the Famennian (Late Devonian) *Costaclymenia* or possibly even the older *Manticoceras* from the Frasnian, but in both cases, no complete fossils of that size have been discovered yet. In the Late Triassic, the genus *Pinacoceras* grew to a conch size of about one meter (reconstructed), but there are also no complete fossils known yet of this size. Even in the Early and Middle Jurassic, ammonoid specimens of over one meter lack solid evidence. By contrast, several Late Jurassic fossils have been excavated and documented that fulfil the megafauna criterion^35,64^. Although puzosiids and some other ammonoids of Cretaceous age reached impressive sizes, species of over one meter conch diameter are the rare exception^38–40,65^. There are a few huge heteromorph ammonoids such as *Baculites*, *Diplomoceras*, or *Emericiceras* (see supplementary Material 5). At least *Baculites* and *Diplomoceras *reached the megafauna threshold size.

## Discussion

### Is there a relation between conch form and size?

**Fig. 5:**
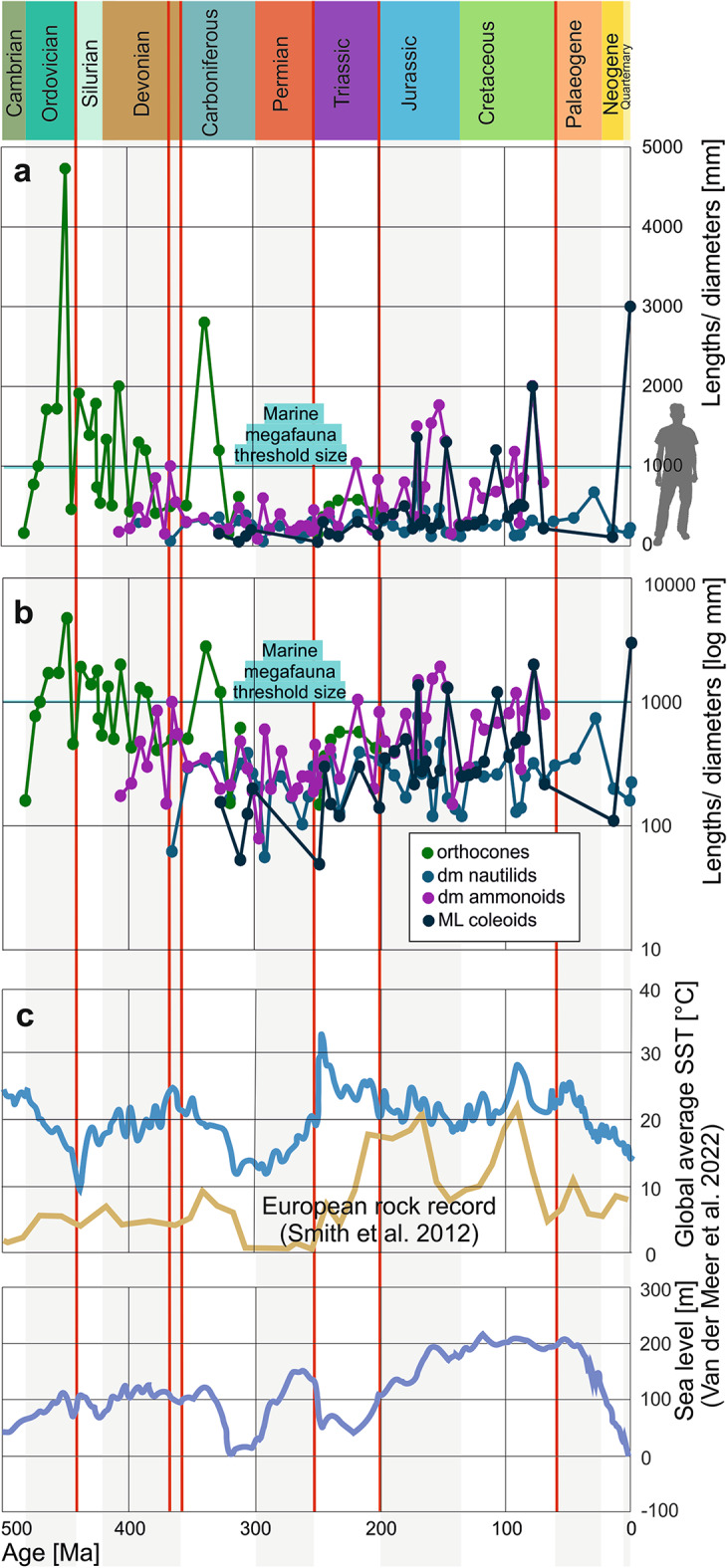
Maximum body sizes in lengths and diameters through the Phanerozoic. **a**, linear plot. Grey silhouette of a 1.8 m tall person for scale. **b**, logarithmic plot. **c**, environmental parameters such as sea-level and sea surface temperatures (blue lines^69^ and the European rock record (brown line^68^). Red lines represent mass extinctions.

**Fig. 6:**
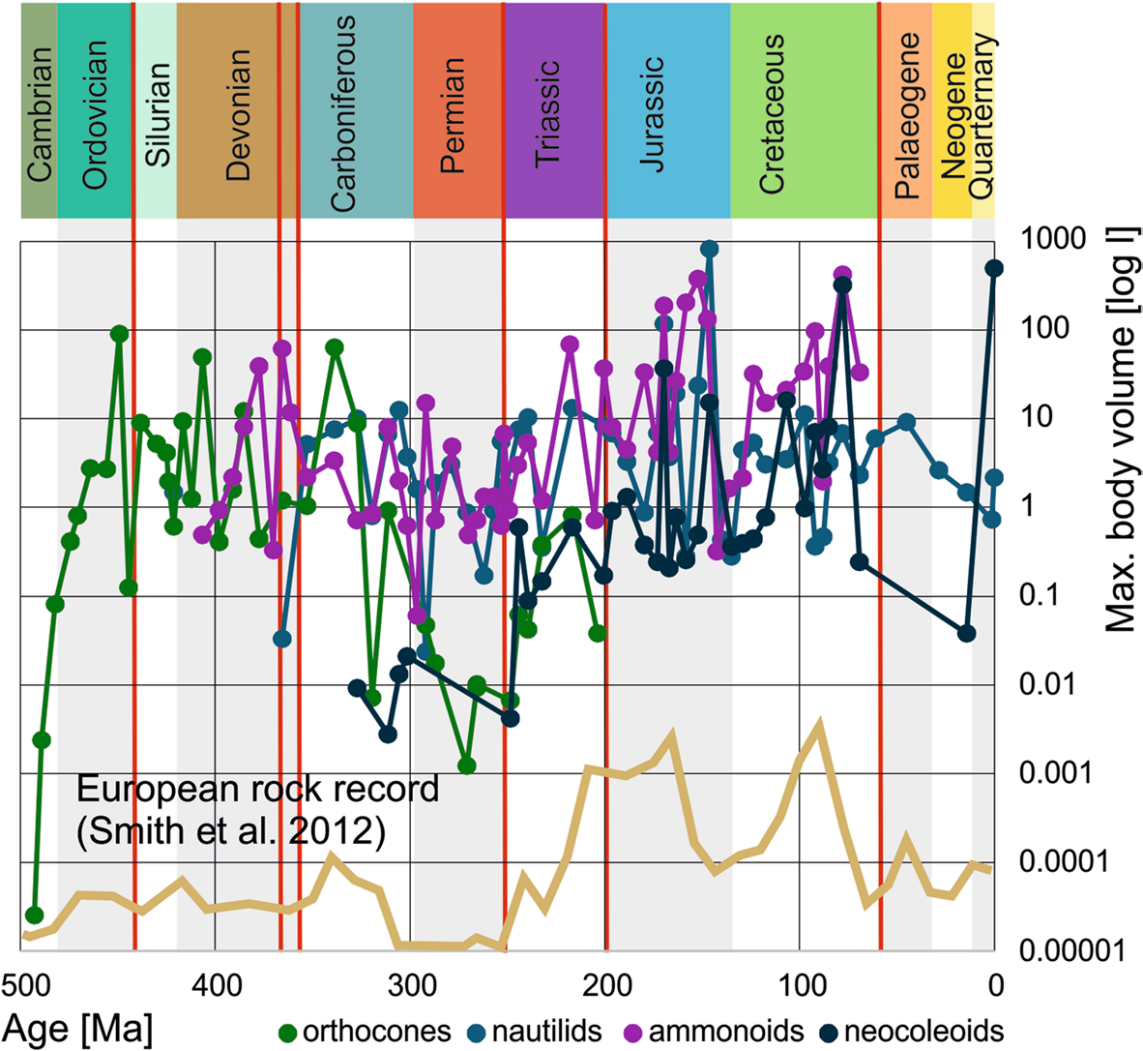
Maximum body sizes in volumes through the Phanerozoic and the European rock record (brown line^68^).

Orthocones: The longest of all cephalopod conchs are found among the endoceratoids (Fig. 5), but in terms of volume, the largest orthocones are in a slightly lower range than the largest representatives of the other three cephalopod groups (Fig. 6). Stevens^35^ estimated that the uncoiled length of the largest ammonoid *Parapuzosia seppenradensis*^38–40^ to amount to an impressive 18.5 m. More precise length data can be derived from a virtually uncoiled ammonoid conch with the help of now commonly applied computed tomography data^66^. Endoceratoids have a rather low apical angle, which explains their extreme lengths at moderate volumes.

Nautilids are rather conservative in both conch size and conch form. This indirectly suggests that conch form may reflect aspects such as metabolic rate, activity level, and thus also conch size (Kleiber’s law^27^).

Concerning ammonoids, it appears to be mostly ‘normal’ ammonoid conch forms^67^, i.e. planispirally coiled forms with moderate whorl expansion rates, whorl width and whorl height indexes, that grew to the largest conch diameter and especially volume (Figs. 1 and 4). Most of the largest ammonoids have a whorl expansion rate between 1.6 and 2.2 and a whorl width-/conch diameter-ratio between 0.2 and 0.3. There are no giants with globular conchs or extremely low whorl expansion rates unless one includes heteromorphs.

Neocoleoids start in the Carboniferous with short mantle lengths of less than 300 mm. This likely roots in the similarly moderate size of their bactritoid ancestors^62^. The Permian neocoleoid record is poor, but in the Triassic, groups such as phragmoteuthids and forms with massively calcified rostra appear (aulacocerids). Only with the Mesozoic, belemnites^14^ and early octobrachians evolved morphologies similar to modern squids. These forms increased in mantle length and body volume already during the Middle Jurassic and an increasing number of megafauna-representatives occur in the Cretaceous and today. It appears likely that with the reduction of the internal shell, body size began to increase in the Jurassic. Most large forms belong to various octobrachian subgroups with some belemnite exceptions^13^.

### Do abiotic environmental factors and rock record correlate with trends in maximum conch size?

The global rock record varies regionally and thereby influences raw diversity data through the Phanerozoic^68^. When we take the quality of our data and the body volume fluctuations into account, it becomes evident that, e.g., in the Permian and Paleogene, data is poor, and the cephalopod volumes appear to be relatively low. Hence, the rock record may also bias our knowledge of maximum body size in cephalopods.

We compared body size and volume data to the sea level and sea surface temperature curve^69^. The sea level and sea surface temperature curves^69^ display plateaus from the Silurian to the middle of the Carboniferous, in the late Permian and from the Early Jurassic to the Palaeogene. There is no simple correlation, but there appears to be a tendency that the largest species occur in phases with rather high sea levels and thus large shelf areas^70^. For example, the Jurassic to Palaeogene plateau is a phase where ammonoids and neocoleoids evolved large conch sizes (Fig. 5). The large endoceratids appeared during a late Ordovician highstand. A special case is the small Early Carboniferous peak in sea-level, when orthocones (here *Rayonnoceras*) reached large sizes before their size decline. Most large orthocones lived in a time with moderately high sea levels during the Ordovician and Silurian (Fig. 5). Cephalopod body size correlates negatively with the δ^13^C and thus perhaps primary productivity^18^. During the low sea level phases of the Late Carboniferous and Triassic^69^, almost no cephalopods reached megafauna dimensions and conch sizes stayed low. These patterns are overlain by other factors such as the mass extinctions. For example, the Cretaceous-Paleogene mass extinction caused a strong decline in cephalopod body sizes and volumes, which is not surprising when taking the severe losses among neocoleoids (belemnite extinction) and the ammonoid extinction into account. Similar effects have been reported from Devonian cephalopods^18^. The recent forms have a special status because they have the largest sample, i.e. a size-related Pull of the Recent effect. It might also reflect our even poorer knowledge of oceanic fauna from deep time compared to modern faunas.

### Are there other drivers of large body size?

We did not evaluate positive drivers such as groups of potential prey organisms or primary productivity. These might be important as food availability is key to reach big body sizes. Further aspects such as physiology and metabolic rates were also not studied but are interesting subjects for future research.

The big mass extinctions affected the trajectories of studied cephalopod groups to varying degrees. We are not discussing diversity changes here, but there are notable changes in body volume (Table 2). However, the four groups behaved quite differently throughout their evolution. For orthocones, the end-Ordovician extinction had the most striking effect, with a strong decline in maximum body volume induced by the demise of most of the Endoceratida. Mass extinctions had no straightforward or only a little effect on nautilid body size, which may as well be considered noise. Ammonoids confirm their reputation as a boom-and-bust-clade: All the big mass extinctions caused a distinct decrease in maximum conch size, partially because smaller forms suffered less, perhaps because they required less resources. Among neocoleoids, only the Cretaceous-Paleogene mass extinction had a strong negative effect on maximum body size. However, their diversity is much lower than that of the ammonoids and thus, the data base is poorer.

**Table 2:** Decrease of maximum cephalopod size (to 50% or less of the pre-extinction maximum body size) following mass extinctions.? – poor data; yes – distinct effect; no – no or insignificant effect; extinction. The effects of mass extinctions can be seen best in Fig. 2.

Event	Orthocones	Nautilids	Ammonoids	Coleoids
End Cambrian	No	Does not apply	Does not apply	Does not apply
End Ordovician	Yes	Does not apply	Does not apply	Does not apply
Kellwasser	?	?	Yes	Does not apply
End Devonian	?	?	Yes	Does not apply
Permian-Triassic	?	No	Yes	?
End Triassic	Extinction	No	Yes	Yes?
End Cretaceous	Does not apply	No	Extinction	Yes

In some cases, the absence of a clear effect of mass extinctions may also be caused by the resolution of our data set, which only takes the maximum body size per stage into account, which is common practice^71^. Compared to benthos, nektic predators such as ammonoids and other cephalopods are considered to recover more rapidly from extinction events^72,73^. Thus, a short drop in maximum body size within a single geologic stage may have been overlooked because of our sampling. Furthermore, the largest individuals are comparatively rare within a community^74^ meaning that maximum body size is considerably affected by uncertainty linked to sampling. To solve these problems, a look at finer temporal scales using population averages may provide further insights. As an example, at a regional level, the average body size of Devonian orthocones typically decreased following several minor and major extinction events^18^.

### Macroecological effect of increasing body size

In many localities, cephalopod fossils occur in great numbers in some strata. An estimate of population size of Devonian ammonoids^75^ demonstrated that cephalopods likely played key roles in marine ecosystems throughout most of the Phanerozoic, as prey and as predators of a broad range of size classes. Importantly, as actively swimming animals, many of which migrating vertically, they also contributed significantly to the biological pump transporting nutrients horizontally across oceans and vertically in the water column^76^. Body size and abundance independently increased throughout much of the Phanerozoic and thus, cephalopods are among the most important organisms that keep deeper marine regions habitable by transporting nutrients (through their metabolism and carcasses). Hence, their protection is of great importance today, in times where big pelagic fishes such as tuna and whales, i.e. marine megafauna, are threatened^26^.

### Macroecological processes

Cephalopod body sizes and volumes through the Phanerozoic have hardly been studied in the past decades. We assembled data sets of the largest specimens of the four groups orthocones, nautilids, ammonoids and neocoleoids we could find for every stage since the late Cambrian, mostly from the literature and museum specimens as well as several new world records such as the largest ammonoids of the Givetian, Frasnian, Griesbachian, Pliensbachian, Bajocian and Bathonian stages. Using various equations and proxies, we estimated body volumes to make the resulting values of these groups comparable.

Size trends through the Phanerozoic vary considerably between the four groups. Following a steep volume increase at the beginning, orthocone volumes then tended to decrease continuously until their demise at the end of the Triassic. Nautilids show the slowest but continuous increase in body size and volumes from the Devonian until today, although the largest species lived during the Jurassic. Ammonoids have a rising trendline from the Devonian to the Cretaceous, which is steeper than that of the nautilids but less steep than that of the neocoleoids, which is not surprising since ammonoids became extinct over 65 million years ago while neocoleoids are the most diverse cephalopod group today. Hence, ammonoids and neocoleoids follow Cope’s rule while nautilids and orthocones do not. Ammonoid size underwent repeated strong fluctuations, which correspond to their diversity fluctuations. Remarkably, the group with the greatest longevity, the nautilids, is also the one with the most continuous size and the only group, which never produced species exceeding one meter in diameter (Fig. 3).

The comparison with abiotic factors suggests that the largest species occurred in times with rather high sea levels and temperatures. However, these times also correlate positively with the times of better rock record. Hence, this correlation must be seen critically, although high sea levels imply broad shelves and potentially good food resources. High temperatures imply lower oxygen levels and thus counter giant growth. Importantly, we can assume an important biomass of cephalopods of the world oceans throughout much of the Phanerozoic. As actively swimming animals, they played a key role vertically transporting nutrients in pelagic areas.

These results show that we just start to understand cephalopod macroecology in deep time. Many more detailed studies are needed to reconstruct some of the factors controlling body size and volume as well as diversity of these cephalopod groups. Also, a better understanding of the phylogenetic relationship between the various cephalopods is a pre-requirement for deeper insights.

## Materials and methods

### Groups

**Fig. 7:**
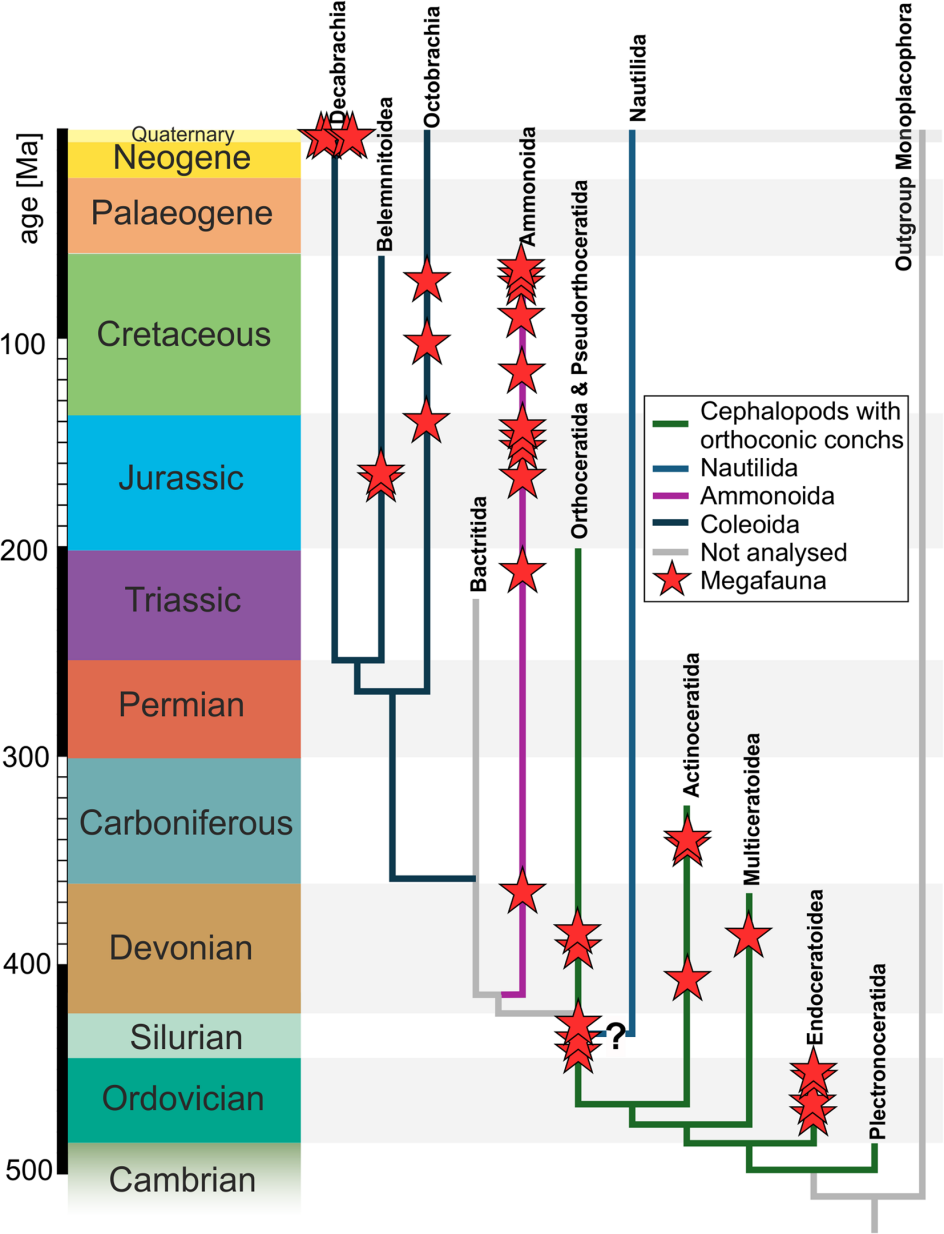
Strongly simplified phylogeny of the Cephalopoda using data published by Kröger et al.^62^ and Pohle et al.^44^ for the early cephalopods with occurrences of megafauna-size cephalopods (red asterisk). Note that all examined groups are monophyletic except the group ‘Cephalopods with orthoconic conchs’.

To achieve a meaningful sample size for each group of cephalopods under consideration that covers a long geologic time interval and comprises at least some diversity, we chose the paraphyletic group ‘cephalopods with orthoconic conchs’ and the monophyletic groups ‘Nautilida’, Ammonoida’, and ‘Neocoleoidea’. We gathered their body size data as follow:

1. Cephalopods with orthoconic conchs (Supp. Material 1, 2): We think that the choice of the paraphyletic group ‘cephalopods with orthoconic conchs’ is justified by their great morphological similarity and therefore presumably similar ecological constraints. It comprises large species of all main clades identified by Pohle et al.^44^, i.e. the Orthoceratoidea, Endoceratoidea and Multiceratoidea (ammonoids or neocoleoids with orthoconic conchs are excluded). Note that this group would be monophyletic when post-Silurian clades are excluded. Our sample of this group includes only forms with more or less longiconic, straight conical conchs to slightly cyrtoconic conchs. The latter are assumed to be reasonably approximated by using a straight conch model as basis for the calculations. This is considered a reasonable compromise as cyrtoconic cephalopods are relatively uncommon, in particular when it comes to large sizes. The amount of the reliable size data is quite inhomogeneous with poor sampling particularly in the Permian and Triassic strata. Palaeozoic data were taken from various sources^17,18^.
2. Nautilida (Suppl. Material 3, 4): This is a monophyletic group. Since the origin of the nautilids is under debate, the delimitation of Nautilida is somewhat unclear^44,77,78^. Sampling is quite good except for a few missing stages. We included only taxa we could confidently assign to the Nautilida starting with the Late Devonian^77^.
3. Ammonoida (Suppl. Material 5, 6): This monophylum^63^ has been partially examined for size previously, but with a focus on Mesozoic forms^35^. The Ammonoida include: agoniatitids, clymeniids, goniatitids, prolecanitids, ceratitids, phylloceratids, and ammonitids (including the lytoceratids). Here, we provide the most complete data set ranging from the Devonian to the Danian^61^, including data of Stevens^35^ and newer studies^17^.
4. Neocoleoidea (Suppl. Material 7): Here, we included the monophylum of Stem and Crown Neocoleoidea *sensu* Hoffmann et al.^63^ excluding ammonoids. This includes all decabrachians, octobrachians and stem neocoleoids. For this group, sampling is poor in the Permian and the Cenozoic. The phylogenetic relationship between these groups is shown in Fig. 7.

### Sampling

We sought the largest individual specimen for each of the four groups listed above per stage (Suppl. Material 8). For the stage age, we used Cohen et al.^79^, the updated version of the international chronostratigraphic chart, version 2024.12. We used the arithmetic mean (i.e. midstage) of the upper and lower age of each stage. In a few cases, we subdivided long stages (Emsian, Famennian). We also plotted the differences of the European rock record through geologic time^68^ to raise awareness of this bias of the fossil record. Indeed, the size data show some positive correlation with that rock record. Since there is also a geographical bias towards records from the global north and particularly Europe, this comparison is meaningful.

### Size and volume

In most cases, lengths and diameters (always in millimetres, where not indicated otherwise) were published, but volume (always in litres) or body mass (always in kilograms) data are available only for recent species. As pointed out in the introduction, the comparability of body length and diameter is limited. This was already highlighted by Stevens^35^, who provided estimates for conch length of species with coiled conchs when the conch was unrolled. This latter approach has the problem that it is difficult to measure. Therefore, we decided to estimate body volume. These are the methods and proxies we used for the four cephalopod groups:

1. Orthocones and neocoleoids with orthoconic conchs (Suppl. Material 1, 2): Because of the simple shape, the conch length l can be approximated for large fragments using the radius r (half diameter d) and apical angle α^17,18^. Suppl. Material 2 is a calculator to determine volumes based on cone length, diameter and apical angle. The length l was determined using the apical angle α and the maximum measured cone diameter (more details in^18^:

l = d_max_/(2 ∙ tan α/2)

The conch volume V was determined using the equation for cones:

V = (1/3) ∙ π ∙ r² ∙ l

**Fig. 8:**
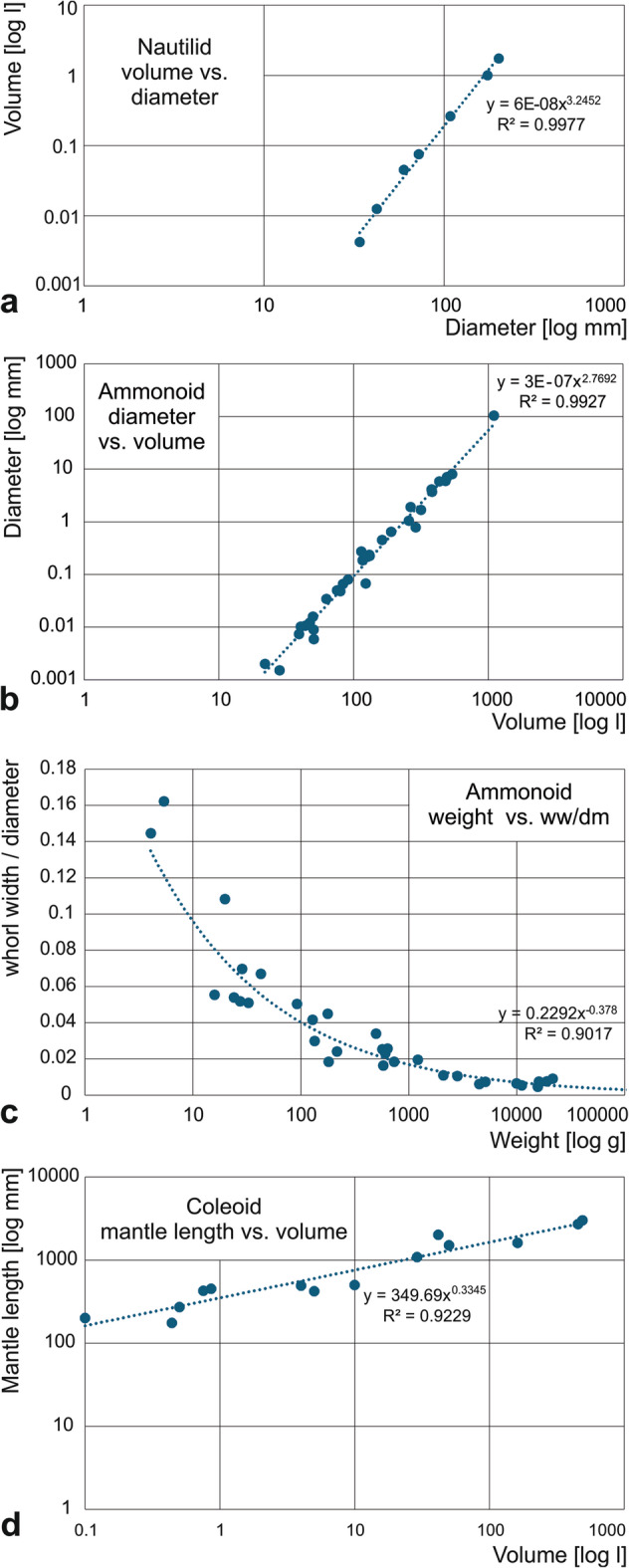
Correlations between parameters that were used to determine volumes. **a**, nautilid volume versus diameter (Suppl. Material 3, 4). **b**, ammonoid volume versus diameter (Suppl. Material 5, 6). **c**, ammonoid body mass versus whorl width-diameter ratio; note that forms with oxyconic conchs fall below the curve and those with thick conchs above (Suppl. Material 5, 6). **d**, neocoleoid volume versus mantle length (Suppl. Material 7, 8); there are stout and slender species, explaining values above and below the trendline.

2. & 3. Nautilids conchs and ammonoids (Suppl. Material 3, 4): In order to obtain an idea of the relationship between conch diameter and volume, we used a series of well-preserved ammonoid and nautilid fossils, which were prepared from both sides. We measured the diameter and the other classical conch parameters^80^. Additionally, we measured the volume directly by submerging the fossils in a measuring cylinder filled with water (water replacement). This approach is only moderately accurate, because the fossils are never perfect. Furthermore, we weighed the specimens and used this mass in combination with rock density to calculate the volume (Suppl. Material 4). We then plotted the obtained volume values (both the directly measured ones and those determined using specimen mass and rock density) of all specimens of the two groups in a log-log-plot and chose the regression line with the best fit (Fig. 8a, b). In both cases, the data points line up well and the R²-value indicated a good value of 0.9927 in the case of ammonoids and 0.9977 in the case of nautilids (Suppl. Material 3, 4).

To obtain an idea of the influence of conch geometry on this diameter-volume relationship, we plotted nautilid and ammonoid body mass (volume) versus the whorl width-diameter ratio (Fig. 8c). The correlation is negative and weakly significant (*p* = 0.058), because the relative whorl width unsurprisingly influences the relation between volume and conch diameter. It is not feasible to obtain accurate volume data of all taxa independently and therefore, we decided to tolerate the error caused by this inaccuracy. This is somewhat justified because most of the large ammonoids included do have a rather ‘normal’ form close to average, i.e. neither globular nor oxyconic (for a graph depicting that error see Fig. 8c). The most extreme exception is the Norian ammonoid genus *Pinacoceras*, which had one of the slenderest conchs with a whorl width-conch diameter-ratio of 0.13 and reaching diameters of over one meter. Still, we consider it reasonable and conservative to use the equation of this regression to reconstruct the volume of those species where we had neither body mass nor volume data:

Volume V = 0.0000003 ∙ dm^2.7692^

We transferred the volume to litres to facilitate imagining the size of the corresponding taxa. This can conveniently be transferred into body mass (biomass) since most of the included cephalopods likely had approximately neutral buoyancy, i.e. the density of sea water (c. 1.03 g/cm^3^ or kg/dm^3^). Here, we consider the biomass of a species as the sum of the masses of all soft parts and all skeletal parts as well as substances held within this body volume including gas, liquids etc.

4: Neooleoids with gladius or pen (Suppl. Material 7, 8): There are almost no 3D-preserved complete soft-body neocoleoids except those from La Voulte-sûr-Rhône, but even those are quite compacted^81,82^ and would not yield reliably correct volume-measurements. Hence, we took literature data of modern neocoleoids and graphed mantle lengths (the standard measurement of neocoleoids) with body mass (Fig. 8d). As in cephalopods with coiled conchs, decabrachian mantle length (ML) turned out to be not perfect but a reasonable proxy for body mass (and thus also volume). The best fitting curve of the two parameters has an R²-value of 0.9229. We thus used its equation to estimate body mass:

Volume V = 349.69 ∙ ML^0.3345^

## Supplementary Information

Below is the link to the electronic supplementary material.

Supplementary material 1 (XLSX 24.2 kb)

Supplementary material 2 (XLSX 10.3 kb)

Supplementary material 3 (XLSX 26.8 kb)

Supplementary material 4 (XLSX 17.4 kb)

Supplementary material 5 (XLSX 35.0 kb)

Supplementary material 6 (XLSX 41.4 kb)

Supplementary material 7 (XLSX 39.6 kb)

Supplementary material 8 (XLSX 66.7 kb)

Supplementary material 9 (DOCX 20.5 kb)

## Data Availability

The literature resources, museum repositories and data needed to evaluate the conclusions in the paper are present in the paper and the Supplementary Materials.
