# Supplementary material for: Cephalopod body size and macroecology through deep time
Source: Sci Rep. 2025 Aug 21;15:30736. doi: 10.1038/s41598-025-13940-1 (PMC12371081; doi:10.1038/s41598-025-13940-1)
Supplement: Supplementary file 9 — Supplementary material 9 (DOCX 20.5 kb) [file 41598_2025_13940_MOESM9_ESM.docx]

**List of Supplementary Materials**

**Supplementary Material 1.** Dimensions, ratios and volumes of large cephalopods with orthoconic conchs per stage including references and data sources.

**Supplementary Material 2.** Excel-sheet with a calculator to determine volumes based on linear measurements.

**Supplementary Material 3.** Dimensions, ratios and volumes of large nautilid conchs per stage including references and data sources.

**Supplementary Material 4.** Volumes and massebody masses of nautilid fossils used to establish a proxy for volume in nautilids.

**Supplementary Material 5.** Dimensions, ratios and volumes of large ammonoid conchs per stage including references and data sources.

**Supplementary Material 6.** Volumes and body masses of ammonoid fossils used to establish a proxy for volume in ammonoids.

**Supplementary Material 7.** Dimensions, ratios and volumes of large neocoleoids per stage including references and data sources.

**Supplementary Material 8.** Sizes and volumes of all cephalopods included in Suppl. data 1, 3, 5, and 7.
